# Profile analysis of college students’ AI literacy and cognitive flexibility and its correlation with deep learning ability

**DOI:** 10.3389/fpsyg.2026.1921158

**Published:** 2026-07-30

**Authors:** Li Simo, Su Lina

**Affiliations:** Department of Humanities, Inner Mongolia University of Technology, Hohhot, China

**Keywords:** AI literacy, cognitive flexibility, college students, deep learning, latent profile analysis

## Abstract

**Introduction:**

AI literacy and cognitive flexibility are essential core capabilities for college students in the age of intelligent technologies. Nevertheless, the heterogeneous group characteristics of college students in these two competencies, as well as their connections with deep learning, have not been sufficiently investigated. This study aimed to classify college students into distinct subgroups according to their levels of AI literacy and cognitive flexibility, explore relevant influencing factors of subgroup categorization, and further clarify differences in deep learning performance across different subgroups.

**Methods:**

A cross-sectional research design was adopted. A total of 591 college students recruited from two universities in mainland China participated in this questionnaire survey. Latent profile analysis (LPA) was used to categorize participants based on AI literacy and cognitive flexibility levels. Multiple logistic regression analysis was applied to analyze demographic and behavioral factors correlated with subgroup belonging. The Bolck-Croon-Hagenaars (BCH) approach was utilized to compare deep learning scores among all identified profiles.

**Results:**

Three distinct latent profiles were extracted: the Dually Constrained group (30.5%), the Latent Potential group (31.3%), and the Comprehensively Integrated group (38.2%). Urban residential background, natural science major enrollment, satisfactory academic performance, and frequent AI application were positive predictors of membership in the Comprehensively Integrated profile. In contrast, rural residency, humanities majors, poor academic achievement, and infrequent AI use were significantly correlated with classification into the Dually Constrained group. Statistically significant upward trends in deep learning scores were observed from the Dually Constrained group to the Comprehensively Integrated group.

**Discussion:**

Obvious heterogeneity exists among college students regarding AI literacy and cognitive flexibility. Students with insufficient AI exposure and unsatisfactory academic performance are more likely to fall into the disadvantaged Dually Constrained subgroup. Targeted educational interventions are required for vulnerable student groups to lift their AI literacy, cognitive flexibility and deep learning outcomes. Notably, given the cross-sectional nature of this research, all detected relationships are correlational rather than causal; causal inference cannot be drawn.

## 1 Introduction

The rapid proliferation of artificial intelligence (AI) technologies has fundamentally reshaped the landscape of higher education, demanding that students acquire not only domain-specific knowledge but also the competencies necessary to navigate an increasingly AI-mediated epistemic environment (Al-Zahrani and Alasmari, 2024; Ouyang et al., 2022; Wang et al., 2025). Among these competencies, AI literacy-broadly defined as the ability to critically understand, evaluate, and ethically apply AI systems-has emerged as a pivotal determinant of students’ adaptive functioning in academic and professional contexts (Lin and Chen, 2024; Tzirides et al., 2024; Wang et al., 2025). Parallel to this, cognitive flexibility, the capacity to shift between cognitive frameworks, tolerate ambiguity, and integrate divergent perspectives, has been consistently identified as a foundational metacognitive resource underpinning effective learning in complex, technology-rich environments (Barak and Levenberg, 2016; Karakuş, 2024; Shah, 2025).

Despite growing scholarly attention to both constructs independently, their co-occurrence and interactive patterning within student populations remain poorly understood. AI literacy and cognitive flexibility are theoretically intertwined: effective engagement with AI tools requires not only technical understanding but also the mental agility to reframe problems, critically interrogate algorithmic outputs, and fluidly adapt to novel informational contexts (Caton et al., 2022). However, prior research has predominantly examined these constructs in isolation, relied on variable-centered analytic approaches, and assumed population homogeneity-thereby obscuring the naturally occurring heterogeneity within student groups.

A person-centered analytic framework offers a methodologically superior alternative, enabling the identification of qualitatively distinct subgroups who share similar configurations across multiple dimensions simultaneously (Morin et al., 2018). Latent profile analysis (LPA), in particular, has been increasingly applied in educational psychology to reveal meaningful typologies of learner characteristics, with demonstrated utility in domains including academic motivation, self-regulated learning, and digital competence (McLarnon, 2024). Yet to date, no study has employed LPA to simultaneously profile students’ AI literacy and cognitive flexibility, leaving critical questions unanswered regarding how these competencies co-develop and cluster across diverse student populations.

A further unresolved question concerns the downstream consequences of such profiling for learning outcomes. Deep learning-characterized by elaborative processing, knowledge integration, and critical meaning-making-represents a hallmark outcome of quality higher education and has been theoretically linked to both metacognitive capacity and digital competence (Jiayuan et al., 2025; Wu et al., 2025; Wu, 2023). If distinct profiles of AI literacy and cognitive flexibility exist, it follows that they may differentially predict students’ propensity for deep learning engagement. Establishing this link would carry significant implications for curriculum design, AI integration strategies, and targeted pedagogical intervention.

Beyond identifying these profiles, it is important to clarify the specific methodological and theoretical value added by the person-centered framework adopted here, relative to conventional variable-centered approaches such as multiple regression or structural equation modeling. Variable-centered techniques estimate a single, population-averaged association between AI literacy, cognitive flexibility, and outcomes such as deep learning, implicitly assuming that this association is uniform across all students. Such models can detect that AI literacy and cognitive flexibility are each associated with deep learning, but they cannot reveal whether students who are simultaneously low on both constructs experience a disproportionate, non-additive disadvantage, nor can they identify naturally occurring subgroups that require qualitatively different forms of support. LPA addresses precisely this gap: by allowing configurations of AI literacy and cognitive flexibility to emerge empirically from the data rather than being imposed *a priori*, it can detect combinatorial or threshold-like patterns-such as the concurrent, mutually reinforcing deficits later observed in the Dually Constrained profile-that a variable-centered model would average away. In doing so, this study resolves a specific and previously unaddressed puzzle: whether AI literacy and cognitive flexibility co-develop in a small number of recognizable configurations among undergraduates, and whether these configurations carry distinct, non-linear implications for deep learning, rather than each competency contributing independently and additively as prior variable-centered studies have assumed.

The present study therefore pursues three interrelated objectives: (1) to identify latent profiles of AI literacy and cognitive flexibility among college students using LPA; (2) to examine sociodemographic and behavioral correlates of profile membership; and (3) to investigate the relationship between profile membership and deep learning performance. By adopting a person-centered, multi-dimensional approach, this study seeks to move beyond aggregate-level descriptions toward a nuanced understanding of how AI literacy and cognitive flexibility jointly structure students’ learning experiences, and to provide an empirical foundation for differentiated, evidence-based educational support.

## 2 Materials and methods

### 2.1 Study design and participants

This study employed a cross-sectional survey design. Participants were recruited from two universities in China between November and December, 2025 using convenience sampling. Eligibility criteria required participants to be currently enrolled full-time undergraduate students with no history of diagnosed cognitive or learning disabilities. A total of 591 valid responses were retained for analysis following exclusion of incomplete or invalid questionnaires.

### 2.2 Measures

#### 2.2.1 Demographic information

A structured self-report questionnaire collected information on gender, region of origin (urban/rural), academic grade (freshman through senior), only-child status, family financial status, academic major (natural science vs. humanities and social science), self-reported academic performance (poor/general/good), and daily frequency of AI tool usage (<1 h/day, 1–3 h/day, >3 h/day).

#### 2.2.2 AI Literacy Scale

Artificial intelligence literacy was measured using the AI Literacy Scale developed by Zhou Qiong and Yingchun (2024), comprising 25 items across four dimensions: AI Knowledge, AI Skills, AI Attitudes and Values, and AI Ethics. Items are rated on a seven-point Likert scale (1 = strongly disagree to 7 = strongly agree); all items are positively scored. Total scores range from 25 to 175, with higher scores reflecting greater AI literacy. Cronbach’s α in the present study was 0.857.

#### 2.2.3 Cognitive flexibility questionnaire

Cognitive flexibility was assessed using the cognitive flexibility questionnaire revised by Wang Yang et al. (2016), which measures individuals’ ability to spontaneously restructure their cognition in response to changing environmental demands. The questionnaire comprises 20 items (e.g., “When explaining why something happened, I consider all the facts and information”) rated on a five-point scale (1 = never to 5 = always). Higher total scores indicate stronger cognitive flexibility. Cronbach’s α in the present study was 0.891.

#### 2.2.4 College Students’ Deep Learning Scale

Deep learning ability was assessed using the College Students’ Deep Learning Scale developed by Lu (2016), comprising 20 items across five dimensions: Practical Reflection, Information Integration, Learning Attitudes, Learning Values, and Comprehension-Based Connection. Items are rated on a five-point Likert scale (1 = completely disagree to 5 = completely agree). Total scores range from 20 to 100, with higher scores indicating greater deep learning ability. Cronbach’s α in the present study was 0.931.

### 2.3 Ethics statement

This study was conducted in accordance with the Declaration of Helsinki. The protocol was approved by the Institutional Review Board (IRB) of Inner Mongolia University of Technology. Written informed consent was obtained from all participants prior to their inclusion in the study.

### 2.4 Data collection

Data were collected between November and December, 2025 using an online self-report questionnaire administered via the Wenjuanxing platform^1^, a widely used survey tool in Chinese educational research. Participants were recruited from two universities located in Neimenggu and Heilongjiang Province, China, through a combination of convenience and cluster sampling. Specifically, academic classes were selected as the primary sampling units, and all students within the selected classes were invited to participate. Research assistants distributed the survey link to class representatives, who then forwarded it to their classmates via WeChat groups. All participants were informed of the study’s purpose, the voluntary nature of their participation, and the confidentiality of their responses prior to completing the questionnaire. A total of 633 responses were collected, of which 591 were retained for final analysis after excluding incomplete responses and those completed in an unreasonably short time (<3 min).

### 2.5 Data analysis

Descriptive statistics were computed for all demographic and study variables using SPSS 28.0. Latent profile analysis (LPA) was conducted using Mplus with full information maximum likelihood (FIML) estimation to accommodate any missing data. Models with two through five profiles were compared using the Akaike Information Criterion (AIC), Bayesian Information Criterion (BIC), sample-size-adjusted BIC (aBIC), entropy, and the Lo-Mendell-Rubin likelihood ratio test (LMR-LRT). Between-profile differences in demographic characteristics were examined using chi-square tests for categorical variables and one-way ANOVA for continuous variables. Multiple logistic regression was performed to examine sociodemographic and behavioral correlates of profile membership. Bolck-Croon-Hagenaars (BCH) method was used to compare deep learning scores across profiles. All tests were two-tailed, and statistical significance was set at α = 0.05.

**FIGURE 1:**
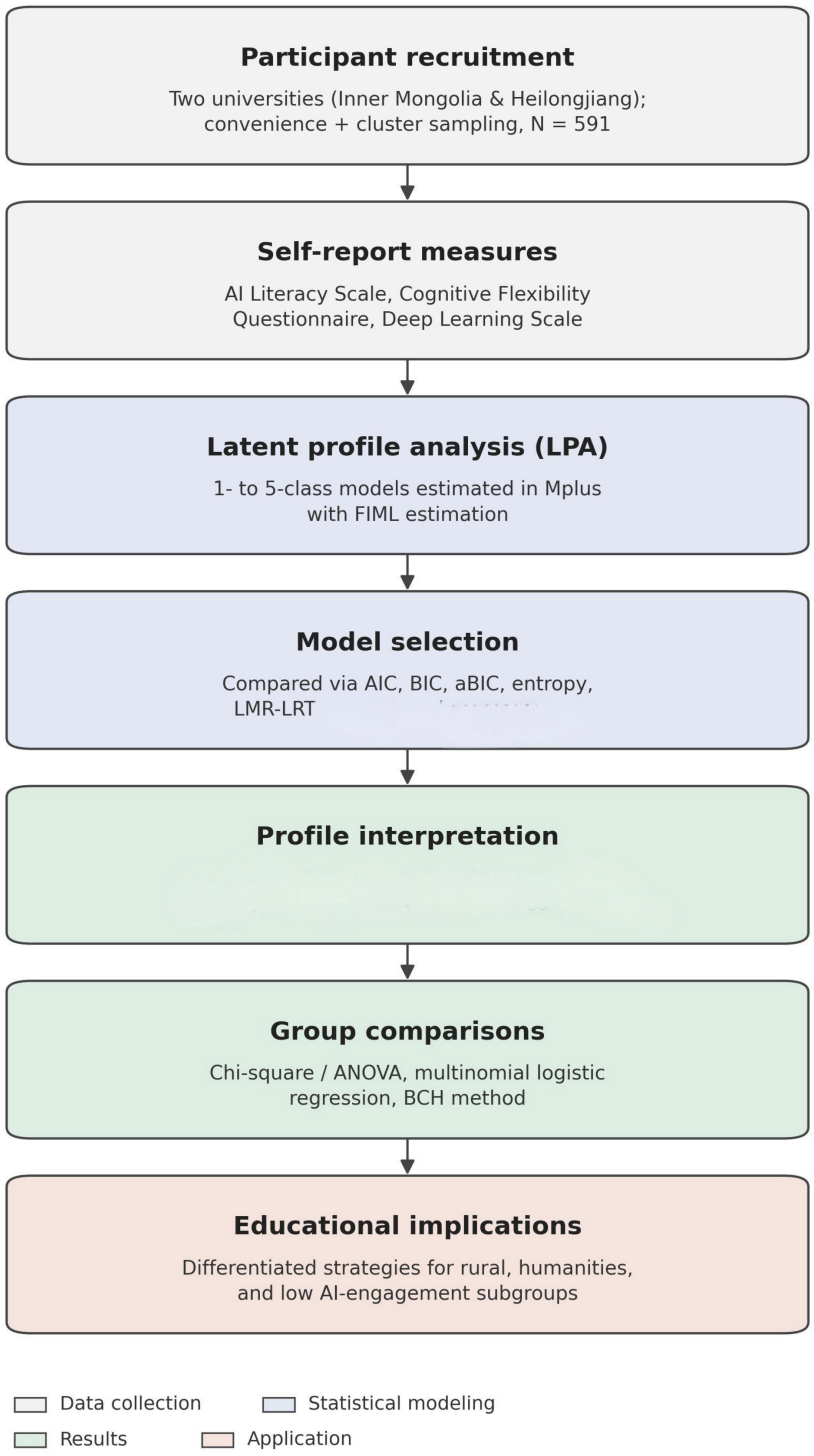
Study design and analytic pipeline.

To enhance the accessibility and reproducibility of the analytic workflow for readers with varying quantitative backgrounds, Figure 1 presents the complete research pipeline, from participant recruitment through latent profile estimation, profile-based group comparisons, and their educational implications.

## 3 Results

### 3.1 Sample characteristics

A total of 591 college students were included in the final analysis, comprising 250 males and 341 females. The majority were urban residents (*n* = 363, 61.4%) and enrolled in humanities and social science programs (*n* = 285, 48.2%). Participants were distributed across all four academic grades, with juniors representing the largest subgroup (*n* = 193, 32.7%). Regarding family structure, 365 participants (61.8%) were single children. In terms of family financial status, the majority reported a general level (*n* = 251, 42.5%), followed by good (*n* = 190, 32.1%) and poor (*n* = 150, 25.4%). With respect to academic major, 306 students (51.8%) were enrolled in natural science programs and 285 (48.2%) in humanities and social science programs. Academic performance was rated as general by the largest proportion of participants (*n* = 294, 49.7%), with 173 (29.3%) reporting good performance and 124 (21.0%) reporting poor performance. Regarding daily AI usage frequency, 278 students (47.0%) reported using AI for 1–3 h per day, 199 (33.7%) for less than 1 h, and 114 (19.3%) for more than 3 h.

### 3.2 Latent profile analysis

Latent profile analysis models with two through five latent profiles were systematically evaluated. Model fit indices are summarized in Table 1. As the number of profiles increased from 2 to 3, the AIC, BIC, and aBIC values decreased substantially, and the LMR-LRT was statistically significant, supporting the superiority of the three-profile solution over a two-profile model. The four-profile solution did not yield a significant LMR-LRT improvement, and one of the emergent classes contained fewer than 5% of the sample, violating the minimum class size criterion. The three-profile model demonstrated an entropy value of 0.968, indicating adequate classification precision. On the basis of these criteria, the three-profile solution was selected as optimal. Details are presented in Table 1.

**TABLE 1:** Model fit indices for latent profile analysis solutions (*N* = 591).

Model	AIC	BIC	aBIC	Entropy	P (LMRT)	P (BLRT)	Probabilities of classes
1	18147.027	18199.609	18161.513	–	–	–	1
2	16466.428	16549.683	16489.364	0.968	0.0000	0.0000	0.60745/0.39255
3	15742.913	15856.840	15774.299	0.968	0.0000	0.0000	0.30457/0.31303/0.38240
4	15747.992	15892.592	15787.828	0.941	0.396	0.407	0.01354/0.28934/0.38240/0.31472
5	15752.695	15927.967	15800.980	0.909	0.491	0.502	0.28765/0.02030/0.01523/0.29442/0.38240

**FIGURE 2:**
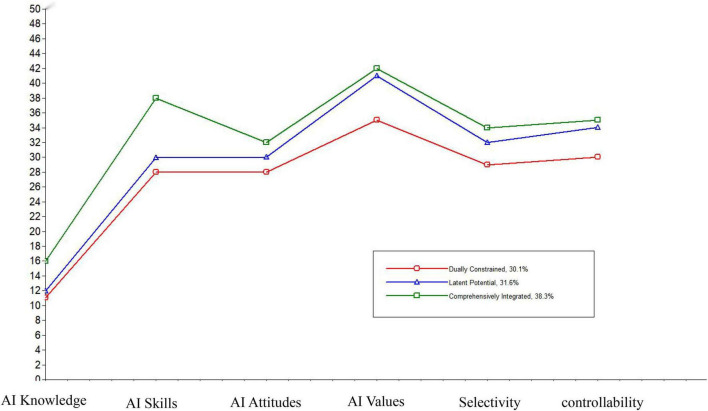
Feature distributions of AI literacy and cognitive flexibility dimensions across the three latent profiles. Class proportions shown in the Figure (30.1%, 31.6%, and 38.3%) reflect model-estimated class probabilities derived from Mplus posterior probability distributions. Class sizes and percentages reported in the main text (*n* = 180, 30.5%; *n* = 185, 31.3%; *n* = 226, 38.2%) are based on modal class assignment. This minor discrepancy is a standard feature of latent profile analysis.

The three identified profiles exhibited distinct and theoretically interpretable patterns across AI literacy and cognitive flexibility dimensions (Figure 2).

Profile 1 - Dually Constrained (*n* = 180, 30.5%): Students in this profile scored consistently low on both AI literacy and cognitive flexibility. The concurrent deficits across both dimensions suggest a fundamental lack of both the technical understanding and the cognitive adaptability required for effective AI engagement. This profile was designated Dually Constrained to reflect the dual nature of the limitations observed.

Profile 2 - Latent Potential (*n* = 185, 31.3%): This profile was characterized by moderate scores on both constructs. Students demonstrated foundational competencies in AI literacy and cognitive flexibility, yet neither had been developed to a level commensurate with high-functioning AI engagement. The designation Latent Potential reflects the developmental capacity apparent within this group.

Profile 3 - Comprehensively Integrated (*n* = 226, 38.2%): Students in this profile exhibited uniformly high scores across all dimensions of AI literacy and cognitive flexibility. This group demonstrated well-developed, mutually reinforcing competencies, reflecting a holistic and integrated mastery of both constructs.

### 3.3 Comparison of general characteristics among college students across different latent profiles of AI literacy and cognitive flexibility

Chi-square analyses indicated significant differences among the three latent profiles in region (χ^2^ = 8.952, *p* = 0.011), academic major (χ^2^ = 9.120, *p* = 0.010), academic performance (χ^2^ = 21.479, *p* < 0.001), and frequency of AI usage (χ^2^ = 15.882, *p* = 0.003), whereas no significant differences were found in gender, grade, single-child status, or family financial status (all *p* > 0.05). Details are presented in Table 2.

**TABLE 2:** Comparison of general demographic characteristics across the three latent profile groups.

	Item	Total	Dually constrained (*n* = 180)	Latent potential (*n* = 185)	Comprehensively integrated (*n* = 226)	X^2^*/F*	*P*
Gender	Men	250	79	71	100	1.703	0.427
	Women	341	101	114	126		
Region	Urban	363	95	125	143	8.952	0.011
	Rural	228	85	60	83		
Grade	Freshman	138	37	40	61	4.731	0.579
	Sophomore	135	43	46	46		
	Junior	193	65	60	68		
	Senior	125	35	39	51		
Single child	Yes	365	119	115	131	2.834	0.242
	No	226	61	70	95		
Family financial status	Poor	150	46	51	53	6.282	0.179
	General	251	80	84	87		
	Good	190	54	50	86		
Major	Natural science	306	78	96	132	9.120	0.010
	Humanities and social science	285	102	89	94		
Academic performance	Poor	124	41	35	48	21.479	<0.001
	General	294	93	111	90		
	Good	173	46	39	88		
Frequency of AI usage	<1 h/day	199	65	64	70	15.882	0.003
	1–3 h/day	278	94	89	95		
	>3 h/day	114	21	32	61		

### 3.4 Multiple logistic regression across different latent profiles

Using the Comprehensively Integrated profile as the reference category, multinomial logistic regression analyses revealed that region, academic major, academic performance, and frequency of AI usage were significantly associated with latent profile membership to varying degrees (see Table 3).

**TABLE 3:** Multiple logistic regression analysis of sociodemographic and behavioral correlates of latent profile membership (*N* = 591).

	Dually constrained					Latent potential				
	β	SE	Wald X^2^	*P*	OR (95% CI)	β	SE	Wald X^2^	*P*	OR (95% CI)
Region										
Rural^a^	−0.458	0.212	4.665	0.031	0.632 (0.417–0.959)	0.208	0.216	0.923	0.337	1.231 (0.806–1.881)
Major										
Natural science^b^	−0.736	0.210	12.267	<0.001	0.479 (0.317–0.723)	−0.327	0.207	2.499	0.114	0.721 (0.481–1.082)
Academic performance										
poor^c^	0.558	0.289	3.727	0.054	1.748 (0.992–3.081)	0.510	0.298	2.933	0.087	1.665 (0.929–2.983)
General^c^	0.765	0.242	9.958	0.002	2.149 (1.336–3.457)	1.086	0.243	20.012	<0.001	2.961 (1.840–4.765)
Frequency of AI usage										
<1 h/day^d^	1.033	0.313	10.920	<0.001	2.810 (1.523–5.186)	0.618	0.286	4.677	0.031	1.855 (1.060–3.247)
1–3 h/day^d^	1.115	0.299	13.925	<0.001	3.050 (1.698–5.478)	0.705	0.272	6.727	0.009	2.023 (1.188–3.446)

^a^Refers to using “Urban” as the reference group.

^b^Refers to using “Humanities and social science” as the reference group.

^c^Refers to using “good” as the reference group.

^d^Refers to using “>3 h/day” as the reference group.

With regard to factors associated with membership in the Dually Constrained profile, region showed a significant association (OR = 0.632, 95% CI: 0.417–0.959, *p* = 0.031), with rural students being significantly less likely than urban students to belong to this profile. Academic major demonstrated the strongest association (OR = 0.479, 95% CI: 0.317–0.723, *p* < 0.001), indicating that natural science students had a significantly lower probability of belonging to the Dually Constrained profile compared to humanities and social science students. Regarding academic performance, students with general performance were significantly more likely to be classified into the Dually Constrained profile than those with good performance (OR = 2.149, 95% CI: 1.336–3.457, *p* = 0.002), whereas the difference between students with poor and good performance did not reach statistical significance (*p* = 0.054). With respect to AI usage frequency, students who used AI for less than 1 h per day (OR = 2.810, 95% CI: 1.523–5.186, *p* < 0.001) and those who used AI for 1–3 h per day (OR = 3.050, 95% CI: 1.698–5.478, *p* < 0.001) were both significantly more likely to belong to the Dually Constrained profile than students who used AI for more than 3 h per day.

With regard to factors associated with membership in the Latent Potential profile, neither region nor academic major reached statistical significance. Students with general academic performance were significantly more likely to belong to the Latent Potential profile than those with good performance (OR = 2.961, 95% CI: 1.840–4.765, *p* < 0.001), while the difference between students with poor and good performance was not significant (*p* = 0.087). Regarding AI usage frequency, students who used AI for less than 1 hour per day (OR = 1.855, 95% CI: 1.060–3.247, *p* = 0.031) and those who used AI for 1–3 h per day (OR = 2.023, 95% CI: 1.188–3.446, *p* = 0.009) were both significantly more likely to be classified into the Latent Potential profile compared to students who used AI for more than 3 h per day.

### 3.5 Profile differences in deep learning

The BCH method was employed to examine differences in deep learning across the three latent profiles. As presented in Table 4, significant differences in deep learning scores were observed among the three profiles (χ^2^ = 33.727, *p* < 0.001). The Comprehensively Integrated profile reported the highest deep learning scores (67.179 ± 0.456), followed by the Latent Potential profile (65.842 ± 0.634) and the Dually Constrained profile (63.063 ± 0.547). Pairwise comparisons indicated that the Comprehensively Integrated profile scored significantly higher than the Dually Constrained profile (*p* < 0.001), and the Latent Potential profile scored significantly higher than the Dually Constrained profile (*p* < 0.001).

**TABLE 4:** Profile differences in deep learning scores (mean ± SE).

Variables	Dually constrained (*n* = 180)	Latent potential (*n* = 185)	Comprehensively integrated (*n* = 226)	*X* ^2^	Pairwise comparison
Deep learning	63.063 ± 0.547	65.842 ± 0.634	67.179 ± 0.456	33.727**	➀< ➂ **; ➀< ➁ **

➀ refer to Dually Constrained group; ➁, refer to Latent Potential group; ➂, refer to Comprehensively Integrated group. No significant difference was observed between ➁and ➂(*p* > 0.05).

**P < 0.001.

## 4 Discussion

The present study employed latent profile analysis to identify three qualitatively distinct profiles of AI literacy and cognitive flexibility among 591 college students, designated as Dually Constrained, Latent Potential, and Comprehensively Integrated. These profiles exhibited differential associations with sociodemographic and behavioral characteristics, and were systematically related to deep learning performance. Collectively, these findings advance understanding of how AI literacy and cognitive flexibility co-configure within student populations, and offer a person-centered empirical foundation for targeted educational intervention.

### 4.1 Interpretation of the three profiles

The identification of three discrete profiles is consistent with theoretical models positing that AI-related competencies and cognitive resources do not distribute uniformly across learner populations, but rather cluster into recognizable typologies reflecting different developmental trajectories (Chee et al., 2025; Hornberger et al., 2023; Ishmuradova et al., 2025). The Comprehensively Integrated profile, representing approximately 38% of the sample, reflects a state in which high AI literacy and strong cognitive flexibility mutually reinforce one another, enabling students to engage with AI tools in sophisticated, critical, and adaptive ways. This synergistic relationship aligns with the cognitive load and adaptive expertise frameworks, which propose that competence is maximized when domain-specific knowledge is integrated with flexible executive functioning (Gkintoni et al., 2025; Hassen, 2025; Meng et al., 2025).

The Dually Constrained profile, comprising nearly one-third of participants, is of particular concern from an educational standpoint. Students in this group exhibited low scores on both constructs simultaneously, suggesting that deficits in AI literacy and cognitive inflexibility may co-occur and potentially exacerbate one another. Prior research has documented that students with limited metacognitive resources are less likely to engage in the exploratory behaviors necessary for developing technology-related competencies (Atchley et al., 2024; Suryahadikusumah et al., 2024), creating a risk of cumulative disadvantage in AI-integrated educational environments. The Latent Potential profile, representing an intermediate state, is theoretically important as it suggests a group of students possessing foundational competencies that have not yet been activated or sufficiently developed—a profile potentially responsive to relatively modest, well-targeted interventions.

This pattern of results illustrates concretely why a person-centered approach was necessary rather than merely preferable. A variable-centered model regressing deep learning on AI literacy and cognitive flexibility as two separate continuous predictors would have estimated their average, additive contributions across the full sample, but it could not have revealed that the co-occurrence of low scores on both constructs-captured empirically by the Dually Constrained profile-corresponds to a distinctly lower deep learning outcome than either deficit would predict in isolation (section “4.3 Profile membership and deep learning”). This combinatorial, subgroup-specific pattern is the central empirical payoff of the person-centered framework, and it directly addresses the previously unresolved question of how AI literacy and cognitive flexibility jointly, rather than independently, structure undergraduates’ capacity for deep learning.

### 4.2 Influencing factors of profile membership

Academic major was significantly associated with Dually Constrained membership, with humanities and social science students demonstrating elevated odds of membership relative to natural science students. This pattern likely reflects differential curricular exposure to computational and analytical thinking rather than any inherent cognitive difference between disciplines. Natural science curricula typically involve systematic engagement with data interpretation, logical reasoning, and increasingly, computational tools, which may incidentally cultivate both AI familiarity and cognitive adaptability (Karampelas, 2025; Park et al., 2023; Waterman et al., 2020). These findings carry direct implications for curriculum design, suggesting that deliberate integration of AI literacy components within humanities and social science programs may help to reduce disciplinary disparities.

The significant role of daily AI usage frequency is among the most practically actionable findings of this study. Across both lower-functioning profiles, reduced AI engagement was associated with substantially elevated odds of non-optimal profile membership. This finding is consistent with the technology acceptance and learning-by-doing literatures, which emphasize that proficiency with digital tools is contingent upon sustained, purposive practice (Falebita and Kok, 2025; Maurat and Jamison, 2025). Importantly, the effect sizes were larger for the Dually Constrained than for the Latent Potential group, suggesting that insufficient AI engagement may be particularly consequential for students who are concurrently cognitively inflexible. Educators and institutions should therefore consider not only whether students have access to AI tools, but whether they are engaging with them with sufficient frequency, depth, and intentionality.

The association between general academic performance and membership in the two lower-functioning profiles, but not between poor and high performance, deserves attention. This non-linear pattern may reflect a “middle-performance paradox,” wherein students with general academic standing- neither sufficiently high to leverage metacognitive resources effectively, nor struggling enough to actively seek support- are particularly vulnerable to stagnation in AI literacy and cognitive flexibility development. This finding suggests that intervention efforts should not be exclusively directed toward academically struggling students, but should also encompass the broad middle tier of the student population.

The differential role of region of origin, which predicted Dually Constrained but not Latent Potential membership, suggests that the urban-rural disparity in AI-related competencies may be most pronounced at the lower end of the competency spectrum. Urban students’ greater prior exposure to AI technologies, more developed digital infrastructure, and broader access to extracurricular technology programs may collectively foster early advantages in both AI literacy and cognitive flexibility (Sanri, 2025). These systemic inequities highlight the importance of equity-oriented policies in AI education that extend beyond urban centers.

These disciplinary and regional disparities underscore the need for differentiated, stratified pedagogical approaches tailored to specific student subgroups. For humanities and social science students, deliberate integration of AI literacy components into existing curricula is warranted—for instance, incorporating AI-assisted tools into qualitative research methods, digital content analysis, and data-driven inquiry projects. Such integration should be scaffolded progressively, beginning with foundational AI concepts and gradually advancing toward critical evaluation and ethical application, allowing students to develop both technical competence and reflective engagement with AI systems. For students from rural backgrounds, equitable access to digital resources and AI platforms remains a structural prerequisite for competency development. Institutional support measures—including subsidized access to AI tools, peer mentorship programs pairing students with differential prior exposure, and targeted AI literacy workshops designed for diverse backgrounds—may help attenuate the structural disadvantages associated with limited prior technological experience and infrastructure.

### 4.3 Profile membership and deep learning

These findings reveal a threshold pattern in deep learning scores across the three profiles. The Dually Constrained group scored significantly lower than both the Latent Potential and Comprehensively Integrated groups. This pattern suggests that the co-absence of AI literacy and cognitive flexibility constitutes a meaningful barrier to deep learning engagement, rather than each competency contributing incrementally and independently to learning outcomes. These findings are consistent with a growing body of literature linking metacognitive competence and digital literacy to elaborative and integrative learning strategies (Ko, 2026). Students in the Comprehensively Integrated profile, possessing both the technical understanding to leverage AI as a cognitive tool and the flexible thinking to integrate diverse information sources, may be better positioned to engage in the critical, synthetic thinking that characterizes deep learning. Conversely, students in the Dually Constrained group, lacking both resources, may default to surface-level processing strategies that prioritize rote memorization over conceptual integration.

These findings have important implications for pedagogical design. Rather than treating AI tools as neutral add-ons to existing instructional practice, educators should recognize that students’ capacity to benefit from AI-enhanced learning environments is itself profoundly shaped by their AI literacy and cognitive flexibility. Instructional approaches that simultaneously cultivate both competencies, for instance, through scaffolded AI-assisted inquiry tasks requiring students to critically evaluate, synthesize, and reflect upon AI-generated content, may be most effective in supporting deep learning across diverse student profiles. Within the person-centered framework employed in this study, the co-occurrence of high AI literacy and high cognitive flexibility in the Comprehensively Integrated profile was associated with the highest deep learning scores, suggesting that these two competencies may operate synergistically rather than additively. From a theoretical standpoint, cognitive flexibility may function as a mediating mechanism: students who develop a sophisticated understanding of AI systems may be best positioned to translate that understanding into deep learning engagement when they simultaneously possess the cognitive agility to reframe problems, critically interrogate AI-generated content, and integrate information across diverse epistemic sources. This mediating pathway would imply that AI literacy interventions may be most effective when paired with instructional strategies explicitly targeting flexible thinking—such as problem-based learning, Socratic dialogue, and structured metacognitive reflection. Future research employing mediation or moderated mediation analysis within longitudinal or experimental designs is needed to formally test and quantify this hypothesized pathway.

### 4.4 Limitations

Several limitations should be considered when interpreting these findings. First, the cross-sectional design precludes causal inference; all associations reported in this study are correlational in nature and must not be interpreted as evidence of causal relationships. Longitudinal designs capable of tracking the co-development of AI literacy, cognitive flexibility, and deep learning over time, as well as experimental or quasi-experimental designs, are necessary to establish directional and causal claims. Future research should consider longitudinal panel studies and mixed-method designs that integrate quantitative survey data with qualitative interviews, enabling a more nuanced understanding of how these competencies develop within individual students across time.

Second, participants were recruited from two universities located in Neimenggu and Heilongjiang Province using convenience and cluster sampling, which may introduce systematic selection bias and substantially limits the representativeness and generalizability of findings to broader Chinese and international student populations. Future studies should employ stratified random sampling across geographically, institutionally, and socioeconomically diverse settings to enhance external validity.

Third, all measures relied exclusively on self-report questionnaires, introducing potential social desirability bias, recall inaccuracy, and common method variance. The absence of objective academic records, behavioral logs, or performance-based assessments represents a notable limitation. Future research should triangulate self-report data with institutional academic records, digital behavioral trace data from AI platforms, and performance-based assessments to improve construct validity and reduce response bias.

Fourth, the study operationalized AI engagement solely through self-reported daily usage frequency, which constitutes an imprecise proxy for meaningful AI engagement. Frequency cannot distinguish between passive, unreflective consumption and purposive, critical use of AI tools; nor does it capture the quality, intentionality, depth, or variety of AI interactions. Future research should develop and validate multidimensional, behaviorally anchored measures of AI engagement that assess purpose, reflective quality, and task complexity alongside frequency.

## 5 Conclusion

This study provides the first person-centered characterization of college students’ AI literacy and cognitive flexibility using latent profile analysis, identifying three empirically distinct and theoretically coherent profiles: Dually Constrained, Latent Potential, and Comprehensively Integrated. Profile membership was differentially associated with academic major, region of origin, academic performance, and AI usage frequency, underscoring the multifactorial correlates of AI-related competency development. Critically, profile membership was systematically associated with deep learning performance in a hierarchical stepwise pattern, establishing the educational significance of these profiles beyond their descriptive value. These findings call for differentiated, evidence-based educational strategies that address the structural and behavioral antecedents of AI literacy and cognitive flexibility, with particular attention to humanities students, students from rural backgrounds, and those with limited AI engagement. Fostering the integrated development of AI literacy and cognitive flexibility—including further investigation of the potential mediating role of cognitive flexibility in translating AI literacy into deep learning outcomes—represents a promising lever for advancing educational quality in the era of intelligent education.

## Data Availability

The original contributions presented in this study are included in this article/supplementary material, further inquiries can be directed to the corresponding author.

## Footnotes

1
www.wjx.cn
